# Somatostatin and the “Small-For-Size” Liver

**DOI:** 10.3390/ijms20102512

**Published:** 2019-05-22

**Authors:** Amelia J. Hessheimer, Lilia Martínez de la Maza, Farah Adel Al Shwely, Arlena Sofía Espinoza, Fabio Ausania, Constantino Fondevila

**Affiliations:** Hepatopancreatobiliary Surgery and Transplantation, General & Digestive Surgery, Metabolic & Digestive Diseases Institute (ICMDM), Hospital Clínic, CIBERehd, IDIBAPS, University of Barcelona, 08036 Barcelona, Spain; lai_lilia@hotmail.com (L.M.d.l.M.); alshwely@clinic.cat (F.A.A.S.); aespinoza@clinic.cat (A.S.E.); ausania@clinic.cat (F.A.); cfonde@clinic.cat (C.F.)

**Keywords:** liver regeneration, liver resection, liver transplantation, portal hypertension, post-hepatectomy liver failure, “small-for-size” syndrome

## Abstract

“Small-for-size” livers arising in the context of liver resection and transplantation are vulnerable to the effects of increased portal flow in the immediate postoperative period. Increased portal flow is an essential stimulus for liver regeneration. If the rise in flow and stimulus for regeneration are excessive; however, liver failure and patient death may result. Somatostatin is an endogenous peptide hormone that may be administered exogenously to not only reduce portal blood flow but also offer direct protection to different cells in the liver. In this review article, we describe key changes that transpire in the liver following a relative size reduction occurring in the context of resection and transplantation and the largely beneficial effects that peri-operative somatostatin therapy may help achieve in this setting.

## 1. Introduction

The liver is an important organ that fulfills vital functions of metabolism, synthesis, storage, and redistribution of carbohydrates, fats, vitamins, and other nutrients [1]. When partially removed or damaged, the liver also has incredible potential for regrowth. It is this last fact that has allowed for the development of modern surgical practices in which a significant portion of the liver is resected or, in some cases, transplanted, largely without major clinical repercussion. There is a limit as to how much liver may be safely left in a particular patient; however, and this limit currently represents the greatest challenge facing both hepatobiliary and transplant surgeons in their attempts to radically remove primary and metastatic liver tumors, as well as to transplant partial liver grafts to treat patients with end-stage liver disease.

In the settings of major liver resection and liver transplantation, in particular using reduced-size livers, there is an acute increase in portal vein flow (PVF) to a mass of liver accustomed to lower flow-per-unit of tissue. This increase in PVF serves as a stimulus for a subsequent series of intrahepatic events that are necessary to achieve liver regeneration and repair. At the same time, if the increase and stimulus are excessive, liver insufficiency and even failure may develop.

“Small-for-size” syndrome (SFSS) (also known as post-hepatectomy liver failure, PHLF, in the context of liver resection) is one of the most feared consequences of partial liver transplantation and major hepatectomy. Characterized by progressive cholestasis, coagulopathy, encephalopathy, ascites, gastrointestinal bleeding and/or renal failure, the development of post-operative SFSS/PHLF is associated with high morbidity and short-term mortality rates of up to 80%. For this reason, careful surgical planning is critical to ensure “safe” graft or remnant liver >25–30% of its pre-operative mass or volume in patients with normal livers or >40% in livers that are cirrhotic, cholestatic, steatotic, or injured by chemotherapy [2,3,4,5].

Somatostatin is a naturally occurring peptide hormone that exerts a number of effects in living organisms, the majority of which are inhibitory. Due to its specific actions in the splanchnic vasculature, it has found a special role in helping to avoid the adverse effects of hyperdynamic splanchnic and portal blood flow in different clinical situations. In the present review article, we discuss the current understanding of key changes occurring in the immediate period following major liver resection and partial liver transplantation and the effects and outcomes that have been achieved following peri-operative administration of somatostatin in these settings.

## 2. Changes Occurring in the “Small-For-Size” Liver

Liver regeneration is a complex and not entirely understood process. In-depth discussion of liver regeneration is beyond the scope of the present manuscript, and we refer the readership to recent and more comprehensive reviews on the topic [6,7]. Briefly, upon reduction of functional liver mass, PVF to the remnant liver increases, exerting a mechanical effect in the hepatic sinusoids. Rapid remodeling of the extracellular matrix (ECM) leads to release and activation of hepatocyte growth factor (HGF), previously produced by hepatic stellate cells (HSCs) and embedded in its precursor form in the ECM [8]. Interaction between HGF and its tyrosine kinase receptor, Met, as well as activation of epidermal growth factor (EGF) receptor occurs very early after partial hepatectomy, indicating the action of preformed ligands (HGF from the ECM, EGF from the portal vein) [6]. Post-hepatectomy increase in PVF also brings a higher volume of metabolites and signaling molecules to the hepatocytes. Elevation in circulating levels of the cytokines tumor necrosis factor-α and interleukin-6 primes hepatocytes to respond to the mitogenic signals of growth factors, including HGF and EGF, as well as transforming growth factor-α, heparin-binding EGF-like growth factor, and amphiregulin, to enter the cell cycle [9,10,11].

In addition to cytokines and mitogenic growth hormones, levels of bile acids also increase rapidly in the liver after partial hepatectomy, and bile acids are necessary for normal liver regeneration to occur. Bile acids bind the farnesoid X receptor (FXR), which not only triggers pro-mitogenic mechanisms but also protects the liver parenchyma against the accumulation of toxic hydrophobic bile acids. Farnesoid X receptor binds both conjugated and unconjugated bile acids and is expressed in hepatocytes as well as the terminal ileum. In hepatocytes, FXR controls the expression of genes involved in the synthesis (e.g., cholesterol 7α-hydroxylase, CYP7A1), conjugation, uptake, and secretion of bile acids [12]. In enterocytes, binding triggers activation of genes involved in bile acid transport and transcription of fibroblast growth factor (FGF) 15 in mice/FGF19 in humans. FGF15/19 reaches the liver through portal circulation and binds the complex of FGFR4 and its co-receptor β-Klotho to suppress the expression of CYP7A1, thereby suppressing further bile acid synthesis [7,13].

In human whole liver transplantation, blood flow to the graft increases upon reperfusion in the recipient [14]. Flows can more than double due to the loss of normal vascular tone and the persistence of abnormal splanchnic hemodynamics. Normal liver tissue is capable of supporting up to between two- and three-times its standard PVF, and this increase is an important stimulus for hepatic regeneration [15,16,17]. Portal vein flow in excess of this threshold; however, can be detrimental to liver function and survival and significantly increases the risk for the development of SFSS/PHLF in the post-operative period [17,18,19,20,21,22]. This is due not only to mechanical shear stress-induced injury to the cells lining the hepatic sinusoids, including sinusoidal endothelial cell (SEC) denudation and dissection of blood into the peri-portal spaces and beyond [21], but also disproportionate restoration of parenchymal and non-parenchymal cells in the regenerating liver.

At the clinical level, little information has been published regarding the histological changes associated with the development of SFSS. Demetris et al. described five cases of SFSS in adult-to-adult living-donor liver transplantation, including three with needle biopsies coinciding with the onset of clinical signs suggestive of SFSS (e.g., cholestasis). In these biopsies, in addition to centrilobular cholestasis, there was some evidence of SEC injury as well as hepatocellular swelling and acute cellular rejection in all cases [23]. Gruttadauria and colleagues also looked at human livers developing SFSS after both partial liver transplantation and extended hepatectomy and observed centrilobular cholestasis, focal endothelial denudation in the portal vein, and immunohistochemical overexpression of Ki-67 [24]. This last finding—overexpression of the cellular proliferation marker Ki-67—is particularly interesting and points to a pattern of excessive regrowth of hepatocytes that predominates at the time of and may ultimately be the cause of liver failure. This theory of excessive hepatocellular regeneration helps to explain the delay typically seen between surgery and clinical signs and symptoms of SFSS (as its presentation in humans is generally subacute) and to provide a unifying pathophysiological basis for “small-for-size” liver failure after both transplantation, where PVF may be hyperdynamic at baseline, and major hepatectomy, where PVF is largely—at least initially—normal.

**Figure 1:**
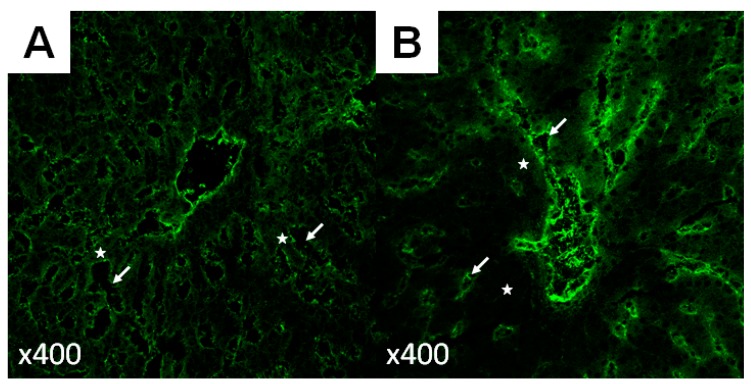
CD31 immunofluorescence of hepatic tissue. CD31, also known as platelet endothelial cell adhesion molecule (PECAM), is expressed in endothelial cells, in the cytoplasm in the case of the hepatic sinusoidal lining. During hepatic regeneration, the replication of hepatocytes precedes that of sinusoidal endothelial cells, leading to the formation of so-called “hepatocyte islands”. Compared to the liver at baseline (**A**), the hepatocyte cords (white stars) in the regenerating liver are thicker and the sinusoids (delimited by fluorescent endothelial cells and marked by white arrows) narrower, with fewer endothelial cells per hepatocyte (**B**).

To understand how hepatocellular regeneration might be “excessive” and how this could be detrimental requires knowledge of how parenchymal and non-parenchymal cells are replaced. Following major size reduction, original liver mass is re-established in a period of weeks in humans, but this does not occur as a balanced process at the cellular level. Timing of regeneration varies according to not only spatial localization but also cell type. Cellular proliferation starts in peri-portal cells (zone 1) and then progresses through midzonal cells (zone 2), and finally reaches the cells closest to the central vein (zone 3). Additionally, during hepatic regeneration, entry into the cell cycle and DNA synthesis occur earlier in hepatocytes versus non-parenchymal cells. For example, DNA synthesis in hepatocytes begins to increase after approximately 12 h and peaks at 24 h in rats (around 36 h in mice), whereas it begins around 48 h in cholangiocytes and Kupffer cells and 96 h in SECs [9,25]. This order of events results in the formation of avascular clusters of 10 to 14 hepatocytes devoid of extracellular matrix, the so-called “hepatocyte islands”, which persist until SECs are replicated and the normal extracellular matrix is restored [26] (Figure 1). Consequently, reformation of the normal liver microarchitecture occurs sometime after the original mass has been replaced.

Hepatocyte islands, which are formed mainly in zones 1 and 2, are considered less functional than normal single-cell-wide hepatocyte plates due to the fact that there are fewer sinusoids and bile canaliculi for each hepatocyte. It; therefore, follows that the smaller the remnant liver, the greater is the proportion of hepatocytes entering the replicative process to form these islands, decreasing the amount of functional liver tissue available even as gross mass is restored [27]. For this reason, several studies have reported that functional regeneration does not correlate with volumetric regeneration during the early stages of liver regrowth [21,28,29,30,31], and that strategies that decelerate regeneration can actually lead to better preservation of normal hepatic microarchitecture and improved survival during the regeneration process [17,22,32].

## 3. Somatostatin Mechanisms of Action

Somatostatin produces a range of actions via binding to the five somatostatin receptor (SSTR) subtypes. Each SSTR associates with heterotrimeric guanine nucleotide-binding proteins (G-proteins) to mediate the inhibition of adenylyl cyclase activity, primarily, as well as mitogen-activated protein kinase and various phosphatases and ion channels [33,34]. Injury, cytokines, growth factors (including insulin and growth hormone, GH), and somatostatin itself regulate SSTR gene expression [35,36,37,38]. Initial responses of SSTR binding are diminished by continuous exposure via mechanisms of receptor desensitization, internalization, and degradation [35,39].

Given that the half-life of somatostatin is very short (1–2 min), it has to be administered as a continuous infusion when applied in vivo. Somatostatin analogues such as octreotide, lanreotide, vapreotide, and pasireotide are more resistant to endogenous peptidases and have significantly longer half-lives than native somatostatin (approximately 2 h for octreotide and lanreotide, 3–4 h for vapreotide, and 12 h for pasireotide) [40]. However, native somatostatin and its synthetic analogues have different affinities for the five SSTR subtypes. Native somatostatin binds all five. Octreotide and lanreotide bind to SSTR2 and SSTR5 and moderately to SSTR3. Vapreotide binds SSTRs 2, 3, and 5 and moderately to SSTR4. Pasireotide has high affinity for SSTRs 1, 2, 3, and 5, making it, in theory, the analogue with the greatest capacity to reproduce the various effects of native somatostatin [41]. Octreotide is the only analogue; however, that has been specifically tested in “small-for-size” livers; while it has never been compared with native somatostatin in this setting, similar effects have been reported for both drugs (see the following section on “Somatostatin and the ‘Small-For-Size’ Liver In Vivo”).

### 3.1. Splanchnic Vasculature

Somatostatin receptors, in particular SSTR2, are expressed on endothelial cells. By binding to its receptors in the portomesenteric vasculature, somatostatin can help decrease portal venous pressure (PVP) and splanchnic blood flow in a dose-dependent manner [42,43]. Somatostatin induces vasoconstriction in the mesenteric arteries and portocollateral veins [44,45]. It also inhibits secretion of gut-derived vasodilatory peptides, such as glucagon, vasoactive intestinal peptide, and substance P [46,47,48]. For this reason, somatostatin has found clinical application in the treatment of acute gastroesophageal hemorrhage in portal hypertensive patients. 

### 3.2. Liver

In the normal innervated liver, somatostatin-containing axons are present in the spaces of Disse and exert paracrine effects on nearby cells, depending on the expression of SSTR subtypes [49]. Receptor expression is limited to cholangiocytes and a small number of endothelial cells lining the hepatic arterioles in the normal liver; after liver injury; however, be it acute or chronic, both hepatocytes and HSCs may express all five SSTR subtypes [22,37,50,51,52].

Once it binds, the action of somatostatin on hepatocytes includes inhibition of hepatotropic factor-mediated hepatocellular proliferation and DNA synthesis when administered prior to partial hepatectomy [53,54,55]. Somatostatin reduces hepatocellular sensitivity to GH by internalizing GH receptors and by decreasing transcription of GH receptor mRNA. As well, it reduces basal and GH-stimulated insulin-like growth factor 1 production in the liver and suppresses the secretion of pro-inflammatory cytokines, many of which are also implicated in hepatic regeneration [34,56,57,58]. The combined effects of somatostatin in the pre-portal vasculature and the liver itself make somatostatin an inhibitor of hepatocellular regeneration. At the same time; however, regenerating hepatocytes may actually down-regulate the expression of somatostatin binding sites, thereby counteracting, at least in part, the suppression of proliferation that somatostatin may exert [53,59]

In addition to hepatocytes, somatostatin may also enact effects on non-parenchymal cells in the liver. While phenotypically injured SECs do not appear to respond to somatostatin directly [22], injured SECs produce endothelin-1 (ET-1), a potent vasoconstrictive peptide that provokes contraction of HSCs. By binding to HSCs (via SSTR1, in particular), somatostatin reduces ET-1-mediated HSC contraction and helps restore the normal hepatic sinusoidal diameter [37].

### 3.3. Potential Side Effects

At standard clinical doses, somatostatin is a relatively safe drug, with few and mostly initial side effects that include nausea, cramps, diarrhea, steatorrhea, and occasionally hyperglycemia [60]. Somatostatin and its analogues have been used for prolonged periods, especially in the treatment of neuroendocrine tumors, and generally appear to be non-toxic [61], though chronic use of second-generation analogues, such as pasireotide, may lead to the development of secondary diabetes. Somatostatin is considered to be the safest therapy for the acute treatment of bleeding gastroesophageal varices [62]. In some animal models; however, acute high-dose therapy has been seen to induce ventilatory depression, blunted responses to hypoxic and hypercapnic stimuli, and even apnea [22,63]. Water intoxication and hyponatremia are also a rare but potentially devastating consequences of somatostatin use [64]. For this reason, patients receiving peri-operative somatostatin infusion should have serum sodium levels checked daily.

## 4. Somatostatin and the “Small-For-Size” Liver In Vivo

Somatostatin and its analogues exert numerous concrete effects in the portal and pre-portal vasculature and liver itself. Results of in vivo studies that have been performed in animals and a few in humans help determine whether, on the whole, the balance of these effects may be beneficial or detrimental in “small-for-size” liver remnants and grafts.

### 4.1. Following Major Hepatectomy

Administration of the somatostatin analogue octreotide following 70% hepatectomy in rats has been shown to reduce the restoration of hepatic mass measured up to two weeks post-operatively. While hepatocellular proliferation is reduced, hepatocyte cords in regenerating livers treated with octreotide maintain a more normal morphology compared with untreated controls. Peri-operative octreotide therapy has also been shown to result in improved hepatic reticuloendothelial system activity, with greater cholangiolar and Kupffer cell proliferation, and less growth of inoculated cancer cells (colonic adenocarcinoma, fibrosarcoma) in regenerating livers [65,66]. Albeit not the objective of its acute administration, somatostatin and its analogues are known to have tumor suppressive effects in both primary and metastatic liver tumors [67,68,69,70,71,72,73], and tumor suppression could potentially be a beneficial collateral effect of peri-operative somatostatin or analogue therapy.

In a more extreme model of 90% hepatectomy in rats, peri-operative octreotide therapy significantly improved liver histology, function, and survival, while simultaneously inhibiting early liver regeneration. This study also determined that octreotide altered the levels of metabolites that participate in or are closely associate with the methionine (Met) cycle, a biochemical reaction that produces S-adenosylmethionine (SAMe), an active methyl residual donor for methyltransferase reactions. In particular, 5’-methylthioadenosine (5’-MTA), a metabolite produced when SAMe is shunted out of the Met cycle to the Met salvage pathway, was significantly increased and was found through exogenous administration to improve hepatic histology and reduce inflammatory cytokines following extended hepatectomy [74]. This preliminary finding is intriguing given the anti-oxidant, anti-inflammatory, and even anti-fibrotic effects that have been ascribed to 5’-MTA [75,76,77].

Somatostatin therapy was shown in a non-survival model of 70% and 90% porcine hepatectomies to significantly reduce post-resection PVF and hepatic venous pressure gradient (HVPG) [78]. In a recent pilot study in humans, somatostatin therapy was initiated intraoperatively following completion of major hepatectomy when the post-resection PVP was >20 mmHg. Somatostatin bolus (250 µg) immediately reduced PVP in the majority of cases and was maintained as a continuous 250-µg/h infusion given over the course of five days. Among ten patients treated in this fashion, three (30%) developed PHLF; one case of PHLF resulted in patient death [79].

While hemodynamic parameters did improve acutely with intraoperative somatostatin therapy, this last case series highlights the importance of initiating somatostatin therapy prior to completing hepatectomy, and not after when considerable liver mass is being removed. At our center, when we are concerned about the risk for PHLF (in general, when we are leaving roughly 30% of pre-operative mass for normal livers or 40% for livers that are cholestatic, steatotic, or have previously undergone extensive chemotherapy), somatostatin is started prior to ligating the main portal vein branch feeding the liver being resected. We give somatostatin as a bolus of 250 μg in patients <65 kg or 500 μg in patients ≥65 kg, followed by a five-day infusion of 250 μg/h. Among major hepatectomy patients treated according to this protocol at our center in recent years, there has been no perioperative mortality, and rates of PHLF grades B (deviation from the regular postoperative clinical pathway managed without invasive treatment) and C (PHLF requiring an invasive procedure) [5] have been 9% and 2%, respectively (unpublished data).

Overall, the experimental and clinical evidence available to date suggests that the perioperative administration of somatostatin or its analogue octreotide following major hepatectomy may suppress regeneration but, at the same time, help achieve more orderly regeneration and reduced injury and inflammation during regeneration via a mechanism that may depend, at least in part, on the Met salvage pathway and the production of 5’-MTA.

### 4.2. Following Liver Transplantation

Mechanical flow derivations, namely portosystemic shunts, have been used to reduce PVF and PVP reaching “small-for-size” liver grafts [17,20]. However, these are relatively permanent solutions for a temporary problem, as the critical period of flow-induced injury is limited [80]. Somatostatin therapy offers a reversible means for reducing portal flow and pressure in the early post-transplant period, while the liver graft is still adapting to the altered physiology in the recipient’s body.

Peri-operative somatostatin therapy has been evaluated in animal models of “small-for-size” liver transplantation. In rat liver transplant recipients, somatostatin given preceding hepatectomy and following reperfusion of reduced-size grafts produced an acute reduction in post-reperfusion PVP. During follow-up, somatostatin-treated recipients had lower serum transaminase and bilirubin levels and reduced intrahepatic expression of ET-1. Heme oxygenase-1 (HO-1) is among the most critical of the cytoprotective mechanisms activated during cellular stress, exerting antioxidant and anti-inflammatory functions, modulating the cell cycle, and maintaining microcirculation [81]; expression of HO-1 was observed to be upregulated in somatostatin-treated grafts. As well, treated grafts had improved hepatic microstructure, less hepatocellular apoptosis, and significantly better survival at the end of seven days in comparison with untreated controls (67% vs. 17%, respectively, *p* = 0.036) [82].

**Figure 2:**
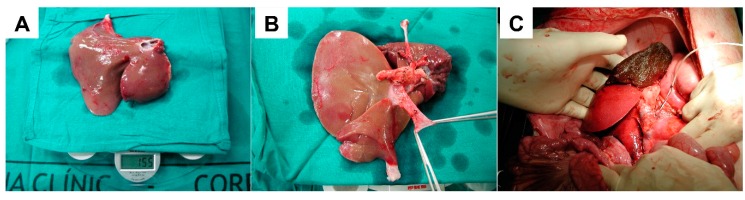
A 70% partial hepatectomy in the porcine model. The left portal vein is ligated, demarcating the plane of transection between the right and left paramedian lobes (**A**). Transection of the hepatic parenchyma is performed using the crush-clamp technique (**B**). Additionally, the lingular projection of the right paramedian lobe is resected (**C**). The remaining 30% of the liver is viewed in situ, with a hemostatic agent applied over the transected surfaces.

Our group has performed “small-for-size” liver transplantation in the porcine model. Seventy percent hepatectomy was performed in donor pigs weighing 15–20 kg, and the reduced-size livers were then transplanted into larger recipients (30–35 kg), avoiding the use of veno-venous bypass by keeping the anhepatic periods short (<20 min) [83]. Transplanted grafts ultimately represented approximately 20% of the recipients’ standard liver volumes (Figure 2). With cold ischemic periods of around five hours and no additional treatment, recipient survival in this model was 26% at five days (5/19 recipients). Hepatic hemodynamic parameters measured in the recipients demonstrated acute increases in PVF and PVP immediately after graft reperfusion, which consequentially produced significant activation and injury at the level of the hepatic sinusoids. Parameters of hepatic regeneration and proliferative activity also rose in direct relation to the degree of post-reperfusion portal hyperperfusion [21]. Perioperative somatostatin therapy, initiated as a bolus during the anhepatic period followed by a continuous five-day infusion, helped significantly reduce PVF and PVP in this model and achieve slower but more orderly hepatic regeneration, better recovery of liver function (uptake, synthesis, and excretion, in particular), and >80% recipient survival at the end of five days [22].

In clinical case reports and series, peri-operative somatostatin treatment has been shown to reduce markers of hepatocellular and SEC injury as well as massive ascites following living donor liver transplantation [84,85,86]. As well, in a recent single-center randomized controlled clinical trial, somatostatin was administered to patients with clinically significant portal hypertension undergoing liver transplantation with whole liver grafts. Patients in the study were randomized 2:1 to peri-operative somatostatin therapy administered during five days (*N* = 18) versus placebo (*N* = 11). The primary endpoint—≥20% reduction in HVPG in response to somatostatin bolus—was met in 55% of somatostatin-treated patients and none receiving placebo. Post-reperfusion HVPG was significantly lower among somatostatin-treated patients, and hepatic artery flows among treated livers were significantly improved. Though the trial was likely underpowered to do so, no differences in major measures of clinical outcome (including adverse events, long-term complications, and graft and patient survival rates) were detected [87].

In liver transplantation, in particular using “small-for-size” grafts, both somatostatin and octreotide produce immediate improvements in portal hemodynamic parameters that translate into less injury, slower but more histologically-normal regeneration, and improved hepatic function in the early post-transplant period.

## 5. Summary and Future Directions

**Figure 3:**
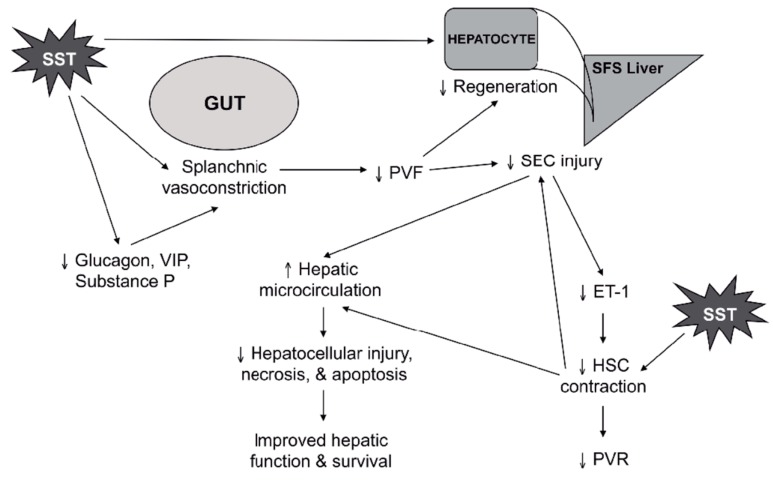
Potential sites and actions of somatostatin (SST) and its analogues in the splanchnic territories and the liver. By binding the endothelium and inhibiting secretion of gut-derived vasodilatory peptides, somatostatin induces vasoconstriction in mesenteric arteries and portocollateral veins and reduces portal vein flow (PVF). In the “small-for-size” (SFS) liver, lower PVF results in decreased sinusoidal endothelial cell (SEC) injury. This translates into reduced expression and translation of endothelin-1 (ET-1) mRNA by SECs. By lowering levels of ET-1 as well as acting directly on hepatic stellate cells (HSCs), somatostatin helps reduce HSC contraction, thereby helping maintain normal hepatic sinusoidal diameter, reducing portal vein resistance (PVR), and improving hepatic microcirculatory flow. Better flow at the microvascular level should lead to less hepatocellular injury and improved function and survival. At the same time, the combined effects of somatostatin on portal inflow and its inhibition of hepatotropic factor-mediated hepatocellular proliferation and DNA synthesis in hepatocytes result in suppression of hepatic regeneration.

Figure 3 and Table 1 summarize the effects that have been observed when somatostatin or its analogues have been administered acutely to “small-for-size” liver remnants and grafts in the context of experimental and a few small clinical trials.

While they decrease regeneration of hepatocytes and the rate of restoration of hepatic mass, somatostatin and its analogues appear to improve the manner in which mass is restored, maintaining a more normal balance between parenchymal and non-parenchymal cells. This appears to translate into improved liver function during the post-operative period and, consequentially, improved survival.

The results of animal and preliminary human studies are promising. However, more clinical work still needs to be done to determine the true extent to which perioperative somatostatin or analogue therapy can prevent and/or reverse pathophysiological processes in “small-for-size” livers and the mechanism(s) by which they may do so, including the impact they may have on the bile acid/FXR/FGF19/FGFR4 and Met salvage pathways. Currently, at least one multicenter randomized controlled trial is underway to determine whether perioperative somatostatin infusion can decrease post-operative ascites following open resection of hepatocellular carcinoma [88], and another trial has been registered to evaluate whether administration of somatostatin can aid in recovery from PHLF that has already been established (NCT02882347, https://clinicaltrials.gov/). In the future, if it is determined that somatostatin or its analogues can safely and consistently help avoid SFSS/PHLF and associated morbidity and mortality, these agents may find greater application in and help expand the clinical indication of major liver resection and partial liver transplantation for the treatment of liver tumors and end-stage liver disease.

## Figures and Tables

**Table 1:** Effects of peri-operative somatostatin or somatostatin analogue therapy on “small-for-size” livers.

Reduces	Improves
Portal vein flow and pressure Generation of ascites Hepatocellular, SEC, and HSC injury Hepatocellular apoptosis Hepatocellular proliferation and restoration of hepatic mass Intrahepatic growth of tumor cells	Preservation of normal hepatic microarchitecture Hepatic reticuloendothelial system activity Hepatic function (e.g., uptake, synthesis, and excretion) Patient survival
